# Dysbindin-1, a Schizophrenia-Related Protein, Functionally Interacts with the DNA- Dependent Protein Kinase Complex in an Isoform-Dependent Manner

**DOI:** 10.1371/journal.pone.0004199

**Published:** 2009-01-14

**Authors:** Satoko Oyama, Hidekuni Yamakawa, Noboru Sasagawa, Yoshio Hosoi, Eugene Futai, Shoichi Ishiura

**Affiliations:** 1 Department of Life Sciences, Graduate School of Arts and Sciences, University of Tokyo, Meguro-ku, Tokyo, Japan; 2 Department of Radiological Technology, School of Health Sciences, Niigata University, Niigata-shi, Niigata, Japan; National Institutes of Health, United States of America

## Abstract

*DTNBP1* has been recognized as a schizophrenia susceptible gene, and its protein product, dysbindin-1, is down-regulated in the brains of schizophrenic patients. However, little is known about the physiological role of dysbindin-1 in the central nervous system. We hypothesized that disruption of dysbindin-1 with unidentified proteins could contribute to pathogenesis and the symptoms of schizophrenia. GST pull-down from human neuroblastoma lysates showed an association of dysbindin-1 with the DNA-dependent protein kinase (DNA-PK) complex. The DNA-PK complex interacts only with splice isoforms A and B, but not with C. We found that isoforms A and B localized in nucleus, where the kinase complex exist, whereas the isoform C was found exclusively in cytosol. Furthermore, results of phosphorylation assay suggest that the DNA-PK complex phosphorylated dysbindin-1 isoforms A and B in cells. These observations suggest that DNA-PK regulates the dysbindin-1 isoforms A and B by phosphorylation in nucleus. Isoform C does not contain exons from 1 to 6. Since schizophrenia-related single nucleotide polymorphisms (SNPs) occur in these introns between exon 1 and exon 6, we suggest that these SNPs might affect splicing of *DTNBP1*, which leads to impairment of the functional interaction between dysbindin-1 and DNA-PK in schizophrenic patients.

## Introduction

Dystrobrevin binding protein 1 (*DTNBP1*, dysbindin-1) consists of approximately 350 amino acids and was originally identified by Benson et al. [1] as a dystrobrevin-binding protein in a yeast two-hybrid screen. Dysbindin-1 attracted interest in 2002 when variations in the gene encoding it at chromosomal locus 6p22.3 were reported to be associated with schizophrenia [2], suggesting a susceptibility locus for schizophrenia. Since then, many groups have reported data that collectively support a link between schizophrenia and *DTNBP1* [3]–[16]. Hence, genetic variations in the dysbindin-1 gene might be a major risk factor for schizophrenia.

Previous reports have shown that diverse high-risk single nucleotide polymorphisms (SNPs) and haplotypes could influence dysbindin-1 mRNA expression [17]–[19]. Moreover, schizophrenics demonstrate reduced dysbindin-1 mRNA expression in the frontal cortex and hippocampus [19], [20], and lower protein expression levels of dysbindin-1 have been observed post-mortem in the hippocampus of schizophrenics compared to age-matched controls [21]. Interestingly, dysbindin-1 is involved in glutamatergic [9], [21] and dopaminergic neurotransmission [22]–[24]. Collectively, this suggests that the physiological function of dysbindin-1 might be impaired in schizophrenia patients. Nevertheless, the functions of dysbindin-1 in the central nervous system (CNS) remain unclear.

To identify the proteins that interact with dysbindin-1, we examined dysbindin-1 binding proteins in lysates from human neuroblastoma cells by glutathione-S-transferase (GST) pull-down assay. We found that the DNA-dependent protein kinase (DNA-PK) complex bound to dysbindin-1 and phosphorylated dysbindin-1 *in vitro*. Interestingly, the functional complex interacted with dysbindin-1 in an isoform-selective manner. Dysbindin-1 isoforms A and B interacted with DNA-PK and localized in the nuclei where DNA-PK complex functions. DNA-PK phosphorylated these isoforms in cells, implying that DNA-PK regulates them by phosphorylations in nucleus. Isoform C does not interact with DNA-PK, not phosphorylated, nor localized in nucleus. These observations suggest a novel function and differences among isoforms of dysbindin-1 in mammalian cells, which could shed new light on the etiology of schizophrenia.

## Results

### Identification of dysbindin-1-associated proteins

**Figure 1:**
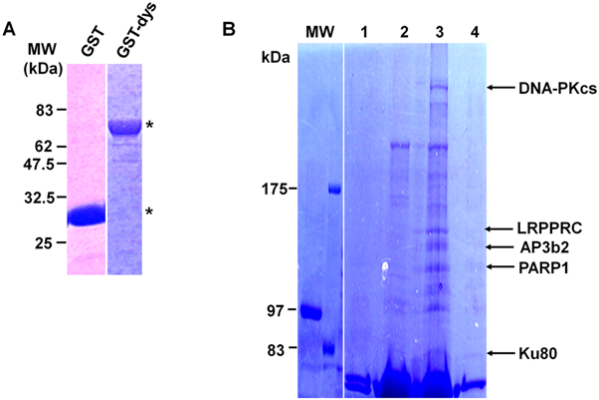
Identification of dysbindin-1-associated proteins. A) Expression of the GST and GST-dysbindin-1 in *E. coli* (asterisk). Purified GST and GST-dysbindin-1 were separated by 10% SDS-PAGE and visualized by Coomassie Brilliant Blue (CBB) staining. B) Proteins identified as dysbindin-1-associated proteins. The purification of GST-dysbindin-1, isolation of lysates from the human neuroblastoma cell line SH-SY5Y, and the pull-down assays were performed as described in the “Materials and Methods.” Eluates of the GST- or GST-dysbindin-1 associated proteins were separated by SDS-PAGE and stained with CBB. Lanes 1 and 2 represent GST and GST-dysbindin-1 alone without cell extract, and lanes 3 and 4 show the associated proteins pulled down by incubating whole-cell extracts from SH-SY5Y cells with GST-dysbindin-1 or GST, respectively. The appropriate portions of the polyacrylamide gel containing the specific protein bands in lane 3 were analyzed by MALDI-TOF-MS as described in the “Materials and Methods.” The arrows indicate the proteins identified by MALDI-TOF-MS with a high score.

To identify proteins that interact with dysbindin-1 in neuronal cells, GST and GST-dysbindin-1 were purified and used to perform pull-down assays in lysates from human neuroblastoma cells (SH-SY5Y), mouse brain, and skeletal muscle. The purities of GST and GST-dysbindin-1 are shown in Fig. 1A. Sodium dodecyl sulfate-polyacrylamide gel electrophoresis (SDS-PAGE) was used to separate the purified proteins, and the gel was then stained with Coomassie Brilliant Blue (CBB). As shown in Fig. 1A, the apparent molecular weights (MW) of GST and GST-dysbindin-1 were 30 and 70 kDa, respectively.

Proteins that were captured by GST or GST-dysbindin-1 were separated by SDS-PAGE and detected by CBB staining. Five proteins, which were ∼80, 115, 120, 157, and 470 kDa, respectively, were co-purified from SH-SY5Y cell lysates with GST-dysbindin-1, but not with GST alone (Fig. 1B). These protein bands were excised from the gel, subjected to in-gel trypsin digestion, and analyzed by mass assisted laser desorption ionization-time of flight-mass spectrometry (MALDI-TOF-MS). They were reproducibly identified as ATP-dependent DNA helicase 2 (Ku80), poly (ADP-ribose) polymerase family, member 1 (PARP1), adaptor-related protein complex 3, beta 2 subunit (AP3b2), leucine-rich PPR-motif containing protein (LRPPRC), and DNA-dependent protein kinase catalytic subunit (DNA-PKcs) by peptide mass fingerprinting (PMF). Interestingly, three of these proteins, Ku80, PARP1, and DNA-PKcs, are components of the DNA-PK functional complex [25], [26]. This suggests that dysbindin-1 might possibly interact with the DNA-PK complex and influence its function. In addition, we also identified several mouse dysbindin-1-associated proteins from mouse brain or skeletal muscle homogenates (Table 1). Previous studies have demonstrated an interaction of AP3b2 with dysbindin-1, which we also identified in screenings of both mouse brain and SH-SY5Y cells. Therefore, we focused on the three components of the DNA-PK complex and investigated their interactions with dysbindin-1 because they revealed the highest PMF score, they are known to physiologically function as a complex, and their interactions with dysbindin-1 have not been defined.

**Table 1:** Proteins identified as dysbindin-1-associated proteins by mass spectrometry.

Bait	Prey	Identified Proteins	NCBI No.
Human dysbindin-1 (isoform A)	SH-SY5Y cells	DNA-PKcs	NP_008835
		LRPPRC	NP_573566
		AP3b2	NP_004635
		PARP1	NP_001609
		Ku80	NP_066964
Mouse dysbindin-1	Brain	Ap3b2	NP_067467
	Skeletal Muscle	Atp2a2	NP_033852
		Atp5a1	NP_031531
		Arfgef2	NP_001078964
		Hadha	AAH37009
		Hadhb	NP_663533
		Dapk1	NP_083929
		Slc25a4	AAH26925

For protein mass spectrometric analysis, GST fusion human dysbindin-1A and mouse dysbindin-1 were used to perform pull-down screening of SH-SY5Y cells, mouse brain, or skeletal muscle. The specific bands from the GST-dysbindin-1 lane were excised and identified by mass spectrometry and peptide mass fingerprinting. The identified proteins from respective bait and preys and their NCBI numbers are shown. These proteins were reproducibly identified by MALDI-TOF-MS with a high score.

### Endogenous dysbindin-1 interacts with Ku70/80 in SH-SY5Y cells

**Figure 2:**
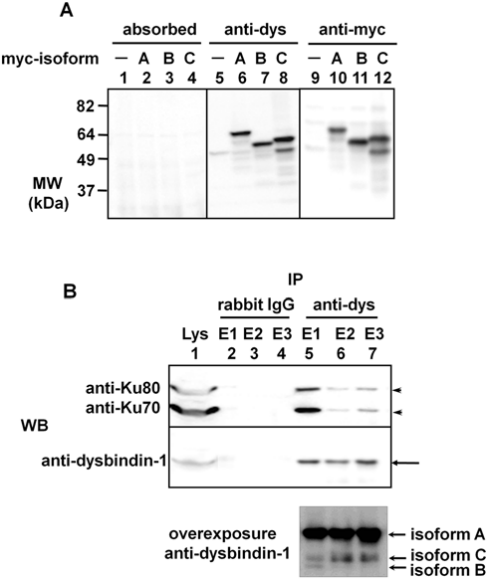
Evaluation of the anti-dysbindin-1 antibody and co-immunoprecipitation of endogenous dysbindin-1 and Ku70/80. A) The anti-dysbindin-1 antibody recognizes all isoforms of human dysbindin-1. The lysates of COS-7 cells overexpressing each isoform were detected by Western blotting using the anti-dysbindin-1 antibody (center panel), the antigen-absorbed antibody (left panel), or the anti-myc antibody (right panel). In the lanes 8 and 12, there were two bands, lower one of which was thought to be the degraded form of isoform C. B) Endogenous Ku70/80 co-immunoprecipitated with endogenous dysbindin-1. Immunoprecipitation was performed using SH-SY5Y cells with the anti-dysbindin-1 antibody or normal rabbit IgG (negative control). As indicated by the arrowheads, Ku70 and Ku80 immunoprecipitated with dysbindin-1 (indicated by arrows), which was pulled down with the anti-dysbindin-1 antibody (lanes 5–7). In the overexposured immunoblot, two more bands corresponding to isoforms C and B were detected, indicating that the protein levels of these isofoms were much lower than that of isoform A.

According to previous studies, it is known that Ku70 forms a heterodimer with Ku80 and is also a component of the DNA-PK complex[27]–[30]. All members of the DNA-PK complex except for Ku70 were identified as binding partners of dysbindin-1, which led us to examine the binding of Ku70/80 to dysbindin-1. To confirm the interaction between endogenous dysbindin-1 and Ku70/80, we generated a polyclonal anti-dysbindin-1 antibody by immunizing rabbits with GST-dysbindin-1 as described in the “Materials and Methods.” Three isoforms of human dysbindin-1 exist in the NCBI database. Hence, the specificity of the dysbindin-1 antibody was evaluated by immunoblotting using lysates from COS-7 cells transfected with myc-dysbindin-1s (isoforms A–C). As shown in Fig. 2A, all three isoforms of dysbindin-1 were detected by the dysbindin-1 antibody (center panel) but not by the antigen-absorbed antibody (left panel). In the absence of myc-dysbindin-1, this antibody reacted weakly with a protein band near the estimated MW of dysbindin-1. The antigen-absorbed antibody did not show immunoreactivity with proteins at this MW. Therefore, we conclude that this band was endogenous dysbindin-1 and used the dysbindin-1 antibody to detect endogenous dysbindin-1 in subsequent experiments.

To ascertain whether endogenous dysbindin-1 interacts with Ku70/80 in neuronal cells, immunoprecipitation assays were performed in SH-SY5Y cells with the anti-dysbindin-1 antibody or with non-specific rabbit IgG (Fig. 2B). The proteins immunoprecipitated by the anti-dysbindin-1 antibody or control IgG were sequentially eluted into three fractions (E1 to E3) and resolved by SDS-PAGE. As shown in Fig. 2B, the anti-dysbindin-1 antibody immunoprecipitated dysbindin-1 and co-immunoprecipitated Ku70 and Ku80; control IgG did not immunoprecipitate dysbindin-1, Ku70, or Ku80. These data suggest that dysbindin-1 might interact with Ku70 and Ku80 in neuronal cells under physiological conditions. However, Ku70 was not identified as a dysbindin-1 binding partner in our pull-down screening. We infer that Ku70 was obscured by the robust dysbindin-1 band because the molecular weight of Ku70 is nearly equal to that of GST-dysbindin-1.

### Localization of endogenous dysbindin-1 in SH-SY5Y cells

**Figure 3:**
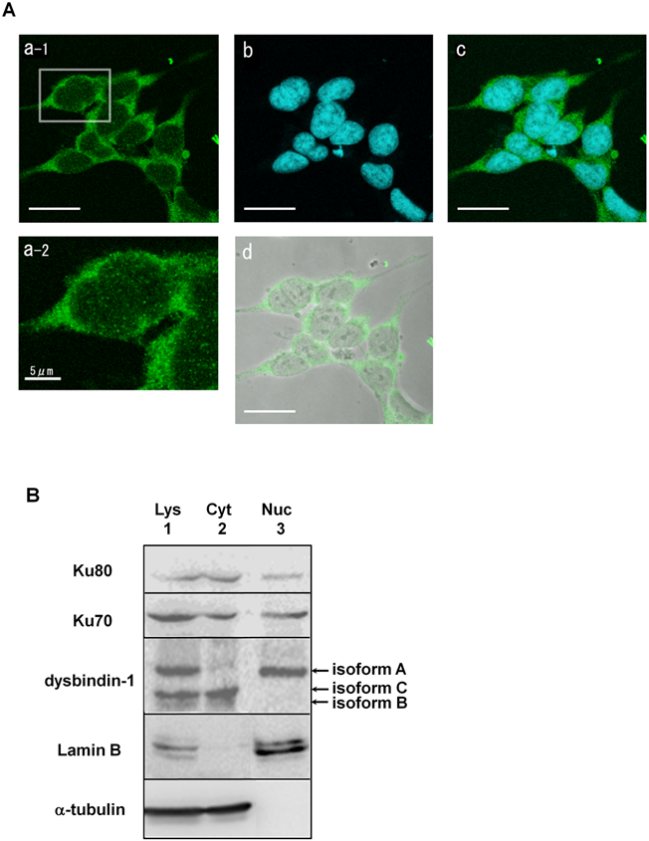
Localization of endogenous dysbindin-1. A) Localization of endogenous dysbindin-1 in SH-SY5Y cells. SH-SY5Y cells were grown on collagen-coated glass coverslips and immunostained using the anti-dysbindin-1 antibody and the secondary antibody conjugated with Alexa 488 (a-1). Nuclei were visualized by incubating with TOPRO3 (b). Nuclei and dysbindin-1 were merged in c. The boxed area is enlarged in the bottom row (a-2), which indicates existence of endogenous dysbindin-1 in nuclei. Green: dysbindin-1; Blue: TOPRO3; White: phase-contrast. Scale bar, 20 µm except for a-2, 5 µm. B) Subcellular distribution of endogenous dysbindin-1 in SH-SY5Y cells. Cytosolic and nuclear fractions were obtained from SH-SY5Y cells and identified with anti-α-tubulin and anti-lamin B antibodies. Lys: whole lysate; Cyt: cytosolic fraction; Nuc: nuclear fraction. Endogenous dysbindin-1 and Ku localized to both the nuclear and cytosolic fractions.

The DNA-PK complex is mainly localized to and functions in nuclei; hence, we examined whether endogenous dysbindin-1 also localized to nuclei in SH-SY5Y cells by immunocytochemistry using the anti-dysbindin-1 antibody. As shown in Fig. 3A, dysbindin-1 appeared to localize primarily to the cytoplasm, but also showed some diffuse localization in the nucleus (Fig. 3A a-2). Phase-contrast imaging showed that these cells were normal, and pre-absorption of the antibodies with antigen GST-dysbindin-1 completely abolished immunoreactivity (data not shown), confirming that endogenous dysbindin-1 exists in SH-SY5Y cells under the physiological condition.

Since this immunocytochemical approach could not clearly show the nuclear localization of dysbindin-1, we performed subcellular fractionation of SH-SY5Y cells followed by immunoblotting. Cytosolic and nuclear fractions were confirmed by immunoblotting using the marker protein antibodies anti-α-tubulin and anti-lamin B, respectively. As shown in Fig. 3B, endogenous dysbindin-1 was unambiguously localized to both the nuclear and cytosolic fractions, which is in accord with our immunocytochemical staining data. We also investigated the subcellular distribution of Ku70/80. Consistent with previous reports [31]–[37], Ku70/80 also localized to both the nucleus and cytosol, supporting the notion that Ku70/80 interacts with dysbindin-1 in neuronal cells. Furthermore, we identified three bands in whole-cell lysates and cytosolic fractions and a single band in the nuclear fraction. The upper, middle, and lower bands are consistent in MW with isoforms A, C, and B, respectively; hence, we hypothesize that isoform A can localize to the nucleus but not isoform C and that it might have a specific biological purpose there.

### Subcellular localizations and interactions with Ku70/80 of three isoforms of dysbindin-1

**Figure 4:**
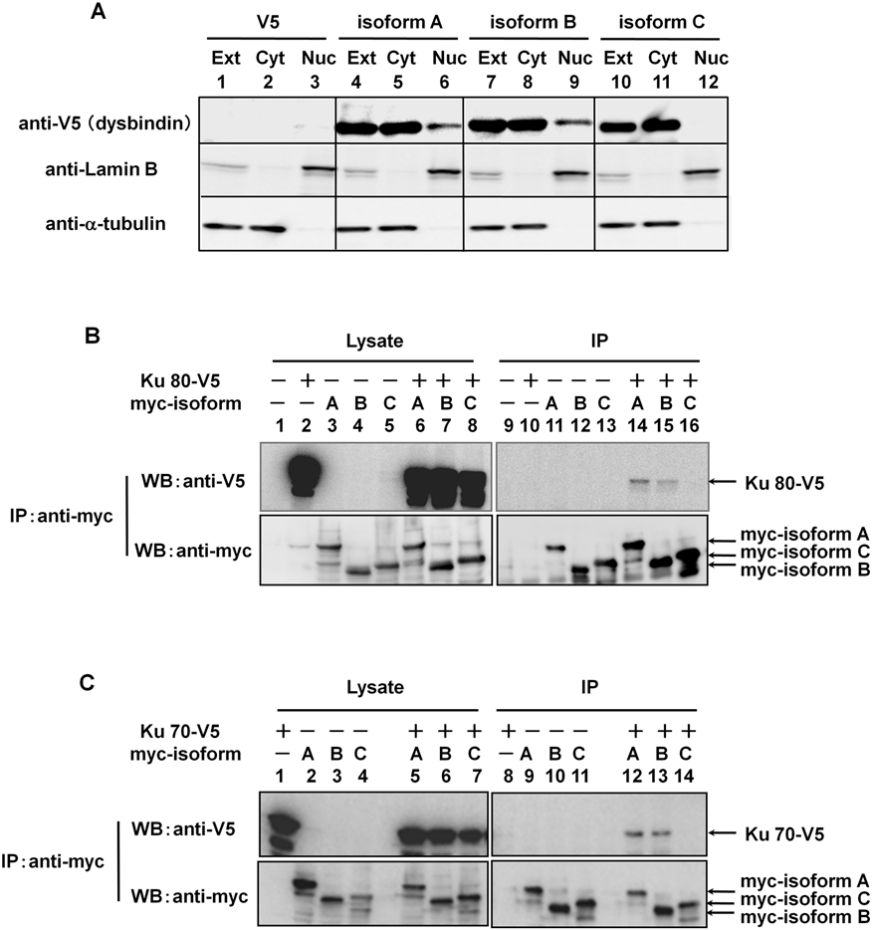
Subcellular localization and interaction with Ku70/80 of three isoforms of dysbindin-1. Protein extracts were prepared from COS-7 cells transfected with the plasmids as indicated. A) Subcellular distribution of three isoforms of dysbindin-1 in transfected COS-7 cells. Cytosolic and nuclear fractions were obtained from COS-7 cells and identified with anti-α-tubulin and anti-lamin B antibodies. Lys: whole lysate; Cyt: cytosolic fraction; Nuc: nuclear fraction. Isoform A and B were localized to both the nuclear and cytosolic fractions but isoform C was not. B) and C) Differences in binding specificities among dysbindin-1 isoforms. Proteins were immunoprecipitated with the anti-myc antibody and detected with the anti-V5 (upper panels) and anti-myc antibodies (lower panels). Ku70/80 were co-immunoprecipitated with isoforms A and B of dysbindin-1 but not with isoform C, indicating that Ku70/80 formed complex with dysbindin-1 in an isoform selective manner.

The subcellular localizations of endogenous dysbindin-1 were different among three isoforms (Fig. 3B). Therefore, to determine the localizations of the isoforms more clearly, we performed subcellular fractionation of COS-7 cells transfected with V5-dysbindin-1A, B and C. The reason why we used COS-7 cells in this overexpression experiment was that the cells were most efficiently transfected. As shown in Fig. 4A, isoforms A and B localized in both cytosol and nucleus, whereas the isoform C was exclusively found in cytosol.

Next, to examine whether Ku70/80 bound to dysbindin-1 in an isoform-selective manner, we performed immunoprecipitation assays in COS-7 cells doubly transfected with respective dysbindin-1 isoforms and Ku70 or Ku80. Both of Ku70-V5 and Ku80-V5 were co-immunoprecipitated with myc-tagged isoforms A and B using the anti-myc antibody, but not with isoform C (Fig. 4B, lanes 14–16, and 4C, lanes 12–14,), suggesting that Ku70/80 bound to isoform-selectively dysbindin-1 in cells. Together with the results of subcellular fractionation (Fig. 4A), these data suggest that only isoforms of dysbindin-1 localized in the nucleus interacted with Ku70/80 there.

### *In vitro* phosphorylation of dysbindin-1 by the DNA-PK complex

We next investigated the functional meaning of binding between dysbindin-1 and DNA-PK complex. As DNA-PK complex is known to be a serine/threonine kinase, we examined whether dysbindin-1 influences DNA-PK kinase activity by comparing the intrinsic kinase activity of the DNA-PK complex in SH-SY5Y cells transfected with myc-dysbindin-1 or empty vector. DNA-PK activities were determined by measuring incorporation of [γ-^32^P] into a synthetic peptide from [γ-^32^P]-ATP by liquid scintillation counting. The intrinsic kinase activity of the DNA-PK complex was not affected by the expression of dysbindin-1 (isoforms A, B, and C) in SH-SY5Y cells (data not shown).

**Figure 5:**
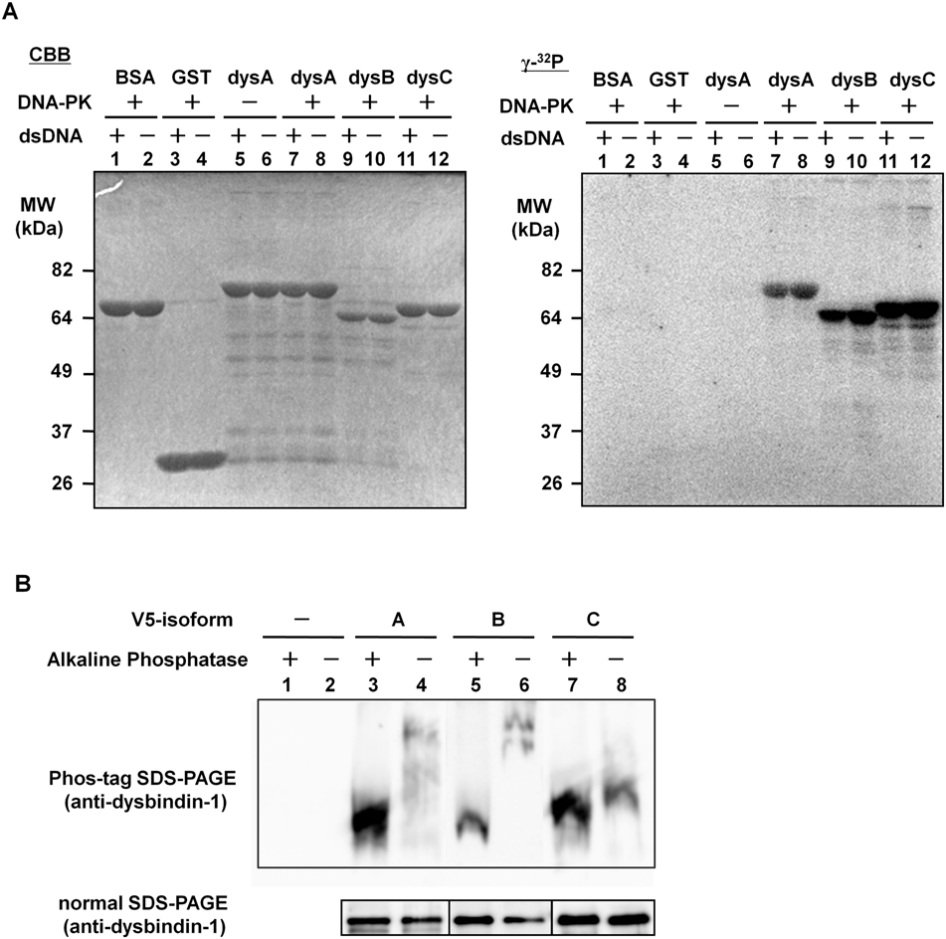
Phosphorylation of dysbindin-1 by DNA-PK complex. A) *In vitro* phosphorylation of dysbindin-1 by DNA-PK complex. The purified DNA-PK complex and GST-dysbindin-1 were incubated with [γ-^32^P]-labeled ATP as described in the “Materials and Methods.” These samples were subjected to 10% SDS-PAGE and stained with CBB (left panel). The right panel shows the uptake of [γ-^32^P] ATP by phosphorylation of dysbindin-1. As shown in the left panel, the amount of BSA (lanes 1, 2), GST (lanes 3, 4), GST-dysbindin-1A (lanes 5–8), B (lanes 9, 10), and C (lanes 11, 12) were equal. Lanes 7–12 in the right panel show that the three isoforms of dysbindin-1 were phosphorylated by the DNA-PK complex. B) Phosphorylation of dysbindin-1 isoforms A and B in mammalian cells. Protein extracts were prepared as described in “Materials and Methods”. These samples were separated by Phos-tag SDS-PAGE and detected with the anti-dysbindin antibody. The phosphorylation levels of V5-dysbindin-1A and B were higher than that of C. Lanes 1 and 2: empty vector; lanes 3 and 4: V5-dysbindin-1A; lanes 5 and 6: V5-dysbindin-1B; lanes 7 and 8: V5-dysbindin-1C. The immunoblot after the normal SDS-PAGE showed that the amounts of dysbindin-1 were not altered after the dephosphorylation procedure by AP.

Next, we investigated whether dysbindin-1 was a substrate for phosphorylation by DNA-PK. We performed *in vitro* kinase assays by mixing the purified DNA-PK complex with GST or GST-dysbindin-1. After the reaction, the samples were subjected to 10% SDS-PAGE, and phosphorylated proteins were detected by incorporation of [γ-^32^P]. As shown in Fig. 5A, all three isoforms of dysbindin-1 were phosphorylated by DNA-PK, whereas BSA, GST, and dysbindin-1 isoform A in the absence of DNA-PK were not phosphorylated. Because DNA-PK activity is influenced by double-stranded DNA (dsDNA) [38]–[41], we next examined DNA-PK activity in the presence or absence of fragmented dsDNA (indicated by + or −). The dsDNA did not affect the phosphorylation level of dysbindin-1 (all isoforms; Fig. 5A), suggesting that phosphorylation of dysbindin-1 might be dependent on the constitutive kinase activity of DNA-PK.

### Phosphorylation of dysbindin-1 in mammalian cells

To examine whether dysbindin-1 is phosphorylated physiologically in cells, we analyzed three isoforms of dysbindin-1 immunopurified from Hela cells transfected with them, using Mn^2+^-Phos-tag SDS-PAGE. The reason why we used Hela cells in this experiment was that the kinase activity of DNA-PK was known to be very high in these cells and most of studies for the DNA-PK function were performed using the cells. As shown in Fig. 5B (lanes 4 and 6), the bands of isoforms A and B up-shifted compared to those of them treated with alkaline phosphatase (AP), indicating that isoforms A and B were predominantly phosphorylated in cells. On the other hand, isoform C were not influenced by the treatment with AP significantly, suggesting that isoform C was phosphorylated at the very low level under the physiological condition. We also observed the same phosphorylation pattern of dysbindin-1 isoforms in COS-7 cells (data not shown). This isoform selectivity of phosphorylation in cells was identical to that of binding to DNA-PK complex and nuclear localization. Combined with *in vitro* phosphorylation data, these observations suggested that DNA-PK complex selectively bound to and phosphorylated isoforms A and B of dysbindin-1, but not isoform C in mammalian cells.

## Discussion

In this study, we identified interaction partners of dysbindin-1 in neuronal cells, mouse brain, and muscle by GST pull-down screening. Dysbindin-1 is a member of biogenesis of lysosome-related organelles complex-1 (BLOC-1) [42] and is thought to be involved in intracellular vesicular trafficking (i.e., protein sorting and vesicle docking and fusion), because genetic deletion of each component of BLOC-1 leads to disruption of intracellular vesicular trafficking in the biogenesis of lysosome-related organelles. All BLOC-1 knockout mice have phenotypes characteristic of Hermansky-pudlack syndrome (HPS), i.e., hypopigmentation of both coat and eyes due to melanosome defects and prolonged bleeding times resulting from platelet dense body defects [42]–[51]. Sandy mice in which dysbindin-1 genes are disrupted also demonstrate such phenotypes and have often been used as HPS model mice [42]. In this study, one of the novel dysbindin-1 binding partners identified was a component of the AP-3 complex, AP3b2 (Table 1). This complex appears to be functionally similar to BLOC-1 because genetic disruption of AP-3 components also results in HPS-like symptoms in mice [48], [52]–[63]. Moreover, physical and functional interactions between BLOC-1 components and AP-3 components were reported recently [64], [65]. Accordingly, we detected the binding of AP3b2 to dysbindin-1, implying that our GST pull-down screening worked well. However, dystrobrevin-1, which was reported to bind to dysbindin-1 in a yeast two-hybrid system [1], was not identified as a dysbindin-1 binding partner. Lack of detection of dystrobrevin-1 in these pull-down assays could be due to the inability to separate dystrobrevin-1 from GST-dysbindin-1 by one-dimensional SDS-PAGE.

Nonetheless, we were interested in the interaction between dysbindin-1 and the DNA-PK complex, which is involved in transcription [66], [67], DNA recombination [68], and DNA repair [38], [40], [69], [70] in many kinds of cells. The physiological role of dysbindin-1 in the nucleus remains to be elucidated, although dysbindin-1 was also reported to localize to nuclei of hippocampal neurons *in vivo* [21]. We demonstrated that both endogenous and exogenous dysbindin-1 bound to components of DNA-PK, Ku70, and Ku80 in neuronal cells by immunoprecipitation, immunocytochemical staining, and subcellular fractionation. Interestingly, dysbindin-1 was also phosphorylated by DNA-PK, suggesting a functional consequence of the interaction. Although phosphorylation of some substrates by DNA-PK is activated by dsDNA [38]–[41], dysbindin-1 phosphorylation by DNA-PK was not affected by the addition of dsDNA, indicating that phosphorylation of dysbindin-1 might not be involved in DNA repair. This is consistent with the idea that DNA-independent DNA-PK activity might also play an important role in transcriptional regulation besides recombination and double-stranded DNA repair as previously described[71]–[73].

Moreover, we found that three isoforms (A, B, and C) of dysbindin-1 interacted with Ku70/80 in different manners in the cells (Fig. 4). Functional differences between these isoforms have not been reported, and the isoform-dependent binding of dybindin-1 to DNA-PK may be of importance. Interestingly, the same isoform-dependency was observed in subcellular localization and phosphorylation in cells. The consistency suggested that DNA-PK complex bound to and phosphorylated dysbindin-1 in mammalian cells as well as *in vitro*. However, our *in vitro* kinase assay data showed that all three isoforms were phosphorylated by DNA-PK (Fig. 5A), implying that all purified isoforms of dysbindin-1 can bind to purified DNA-PK. We hypothesized that the distribution of dysbindin-1 in cells might be different among the isoforms and that localization differences could be the basis of their isoform-dependent interaction properties. Correspondingly, isoforms A and B were detected in considerable amounts in the nuclear fraction (Fig. 3 and 4). Hence, nuclear localization of isoforms A and B might facilitate their binding to Ku70/80. In contrast, isoform C, although abundant in total cell lysates, was not detected in the nuclear fraction. As isoform B appeared to be a minor dysbindin-1 according to its very low expression level in neuronal cells, the functional difference between isoform A and C might be of importance in terms of the physiological roles of dysbindin-1 in CNS.

**Figure 6:**
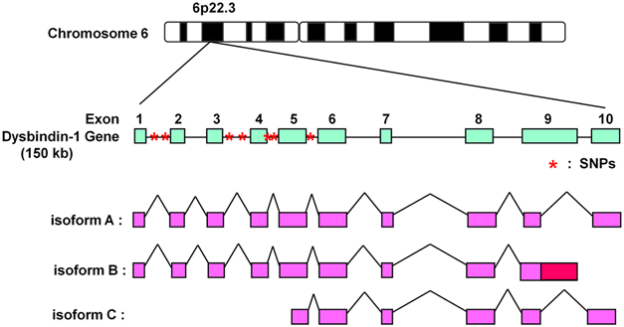
Schematic representation of the human dysbindin-1 gene and its three isoform structures. The gene encoding dysbindin-1 located at chromosomal locus 6p22.3, and its several genetic variations are associated with schizophrenia. These variations (SNPs and haplotypes) are located in intron or promoter regions, and almost all are located in the N-terminus of the gene. Red asterisks indicate major SNPs. Three isoforms (A, B, and C) of human dysbindin-1 were reported by NCBI. Isoform A encodes the longest isoform, and isoform B contains an additional segment in the coding region compared to isoform A. Isoform C contains an alternate splice site in the 5′ coding region and uses a downstream start codon, compared to isoform A; this isoform has a shorter N-terminus compared to isoform A.

What is the difference between dysbindin-1 isoforms A and C? Figure 6 shows the amino acid sequences of the spliced isoforms of dysbindin-1. It appears that isoform C is an N-terminally truncated form of isoform A. Hence, the N-terminal region of isoform A could possibly localize to nuclei and bind Ku70/80. To our surprise, almost all of the schizophrenia-related SNPs are found in the introns between exon 1 and exon 6, which are in isoforms A and B but not in C (Fig. 5B) [2]–[8], [10], [12]–[14], [17], [18]. This led us to hypothesize that the schizophrenia-related SNPs in the coding region of the dysbindin-1 gene might affect its splicing variations and lead to a reduction in isoform A and an increase in the content of isoform C, which could be defective with regard to interaction with the DNA-PK complex.

In conclusion, in this study we describe for the first time a functional interaction between dysbindin-1 and the DNA-PK complex and show a functional difference in dysbindin-1 isoforms. This newly acquired information provides a basis for the novel hypothesis that alternative splicing of dysbindin-1 due to schizophrenia-related SNPs may underlie the etiology of schizophrenia. Further studies that focus on the relationship between schizophrenia-related SNPs and dysbindin-1 splice variants and the expression levels of the three isoforms in the brains of post-mortem schizophrenic patients would be helpful in understanding the role of dysbindin-1 in schizophrenia.

## Materials and Methods

### Plasmid Construction

Three isoforms of human dysbindin-1 cDNA and mouse dysbindin-1 cDNA were cloned from a human fetal brain cDNA library (BD Biosciences) and a mouse brain cDNA library by polymerase chain reaction (PCR), respectively. These cDNAs were subcloned into pcDNA3.1 (Invitrogen) or pGEX-4T-3 (GE Healthcare), which contained His-tag×6 at the C-terminus. The myc-dysbindin-1 contained myc-tag×6 at the N-terminus of dysbindin-1, and V5-dysbindin-1 contained a V5-tag at the C-terminus of −1. GST-dysbindin-1 was able to express GST protein at the N-terminus and His-tag×3 at the C-terminus of dysbindin-1. The full-length human Ku80 and Ku70 were cloned from a human fetal brain cDNA library by PCR. The cDNAs of Ku80 and Ku70 were subcloned into a pcDNA3.1 expression vector that contained a V5-tag in the C-terminus of the cDNA. All constructs were confirmed by sequencing using a fully automated DNA sequencer (Beckman Coulter).

### Cell Culture and Transient Transfection

The cell lines SH-SY5Y and COS-7 were cultured in Dulbecco's modified Eagle's medium (Sigma-Aldrich) supplemented with 10% fetal bovine serum (Invitrogen) in a 5% CO_2_ atmosphere at 37°C. SH-SY5Y cells were plated in collagen-coated dishes and were harvested at 100% confluency for GST pull-down assay and immunoprecipitation analysis. COS-7 cells were transiently transfected with plasmid constructs using FuGENE 6 transfection reagents (Roche Applied Science). After 48 h of transfection, the cells were harvested for immunoprecipitation.

### Expression and Purification of GST and GST-dysbindin-1

A pGEX expression vector containing human (isoform A) or mouse dysbindin-1 was transformed into BL21 (DE3) strain. An overnight culture of the transformant in Luria Broth (LB) medium was diluted and shaken at 37°C until the OD_600_ reached 0.3–0.5; 0.1 mM isopropyl-β-D-thiogalactopyranoside (IPTG) was then added. During induction by IPTG, the culture was shaken at 27°C for 3 h. The bacterial cells were collected by centrifugation (1,600×*g* for 20 min), washed with phosphate-buffered saline (PBS), and suspended in sonication buffer [50 mM Tris-HCl (pH 7.0), 200 mM NaCl, 1 mM EDTA, 1 mM DTT, 0.2 mM PMSF, and 1/1000 volume of protease inhibitor cocktail (Sigma-Aldrich)]. The suspended *Escherichia coli* were fractured four times by a FRENCH® pressure cell press (Ohtake Works, Co.), and Triton-X 100 was added to a final concentration of 1%. The suspension was then incubated for 30 min on ice. The lysates were centrifuged (16,000×*g* for 30 min), and the supernatant was subjected to affinity purification using glutathione Sepharose 4B beads (GE Healthcare) and Talon Metal Affinity Resin (BD Biosciences), according to the manufacturer's protocol. All subsequent steps were performed at 4°C. The quantity and purity of the proteins was assessed by SDS-PAGE; Coomassie Brilliant Blue (CBB) was used to stain the separated proteins.

### GST Pull-Down Assay

The SH-SY5Y cells were homogenized in lysis buffer [50 mM Tris-HCl (pH 7.5), 150 mM NaCl, 1 mM EDTA, 1% (w/v) Triton-X 100, 0.2 mM PMSF, and 1/1000 volume of protease inhibitor cocktail] and incubated for 30 min on ice. Mouse brain or skeletal muscle (2 g each) was homogenized in lysis buffer using a Hitachi homogenizer and incubated for 30 min on ice. The lysate was centrifuged (16,000×*g* for 30 min), and the supernatant was precleared with glutathione Sepharose 4B beads for 1 h. The precleared lysate was incubated with equivalent amounts of GST and GST-dysbindin-1, respectively, for 3 h at 4°C. After incubation, the beads were washed four times with lysis buffer, and the bead-bound proteins were eluted by boiling for 5 min in 2×SDS sample buffer. The precipitated proteins were separated by SDS–PAGE, and the specific bands in the GST-dysbindin-1 lane were analyzed by MALDI-TOF-MS.

### Protein Identification by Mass Spectrometry

Following electrophoresis, proteins were stained with colloidal CBB. The protein bands of interest were excised from the gel, cut into small pieces, dehydrated with acetonitrile (ACN) for 10 min, and dried completely in a vacuum centrifuge. DTT-containing buffer (10 mM DTT in 100 mM NH_4_HCO_3_) was added to the gel pieces, and the gel pieces were incubated for 1 h at 56°C. After the samples had cooled to room temperature, the DTT-containing buffer was replaced with iodoacetamide-containing buffer (55 mM iodoacetamide 100 mM NH_4_HCO_3_), and the gel pieces were vortexed for 45 min at room temperature. The gel pieces were then washed with 100 mM NH_4_HCO_3_ buffer and dehydrated by ACN several times repeatedly and dried in a vacuum centrifuge. The gel pieces were then incubated in trypsin (Promega)-containing buffer (12.5 ng/µL trypsin in 50 mM NH_4_HCO_3_ and 5 mM CaCl_2_) in an ice-cold bath. After 45 min, the protein digestion was performed overnight at 37°C. Digestion was stopped by the addition of 0.1% TFA in 50% (v/v) ACN/water. Peptides were extracted by the addition of 50 mM NH_4_HCO_3_ in 50% ACN, with three changes (20 min per extraction) at room temperature, and concentrated. The peptides in the extract were purified from the supernatant by absorption onto ZipTipC18 (Millipore) according to the manufacturer's instructions. After five washes with 0.1% TFA in water (v/v), bound peptides were eluted with 10 µL of saturated matrix-solution (R-cyano-4-hydroxy-cinnamic acid, Sigma-Aldrich) in 0.1% TFA (v/v) in 75% (v/v) ACN/water. Then, 0.3 ml of each eluted sample were spotted on the target plate repeatedly and dried at room temperature. MALDI-TOF-MS was performed on an AXIMA-CFR mass spectrometer (Shimadzu). MALDI peptide spectra were calibrated using several peaks of self-digested trypsin and matrix ion as internal standards. The data were analyzed using the MASCOT search program (Matrix Science, London, UK). The peptide masses were compared to the NCBI database for identification of the intact proteins.

### Antibodies

The rabbit polyclonal antibody to dysbindin-1 was generated by injecting rabbits subcutaneously with 1.8 mg of purified GST-dysbindin-1 (mouse) protein from *E. coli* using the standard immunization protocol. The antiserum was immunoaffinity-purified using a column in which MBP (maltose binding protein)-dysbindin-1 (mouse) proteins were coupled using Affi-Gel® 15 (Bio-Rad) according to the manufacturer's protocol. Cyclic incubation of the IgG fraction of the antiserum overnight was followed by the elution of affinity-purified antibodies with 100 mM glycine-HCl (pH 2) and neutralized with 1 M Tris-HCl (pH 9). Affinity-purified antibodies were supplemented with 10 mM NaN_3_, stored at 4°C, and diluted 1∶1000 for immunoblotting. The anti-Ku70 and Ku80 antibodies were gifts of Dr. Y. Hosoi, Niigata University. The following mouse monoclonal antibodies were purchased from the vendors indicated in parentheses: anti-myc, anti-V5 and anti-lamin B (Invitrogen) and anti-α-tubulin (Sigma-Aldrich). The followning rabbit polyclonal antibodies were purchased from the vendors indicated in parentheses: anti-V5 (Millipore) and anti-myc (cell signaling).

### Immunoblotting

Samples were separated by SDS–PAGE and transferred to PVDF membranes (Immobilon-P; Millipore). The membranes were blocked with 5% skim milk in PBS with 0.05% Tween®20 (TPBS) for 1 h at room temperature and then incubated for 2 h at room temperature or overnight at 4°C with primary antibodies in 5% skim milk. After washing, the membranes were incubated for 45 min with horseradish peroxidase (HRP)-conjugated secondary antibodies (Cell Signaling) at room temperature. The immunoreactive bands were visualized by enhanced chemiluminescence (ECL) and scanned by LAS 3000 (Fuji film co. LTD).

### Immunoprecipitation

COS-7 cells were transfected with myc-dysbindin-1 and V5-tagged constructs of Ku70 or Ku80 using FuGENE 6. Cells from 10-cm plates were homogenized in 1 ml of lysis buffer [50 mM Tris-HCl (pH 7.5) containing 150 mM NaCl, 1 mM EDTA, 1% (w/v) Triton X-100, 0.2 mM PMSF, and protease inhibitor cocktail]. The lysates were then centrifuged at 16,000×*g* for 30 min at 4°C. The supernatant was precleared with protein G Sepharose 4 fast flow beads (GE Healthcare) for 1 h, and then incubated with 1 µl of anti-myc or V5 antibody for 2 h. The beads (15 µl) were added to the lysate and incubated for 1 h. After the beads were washed four times with lysis buffer, the precipitates were analyzed by SDS–PAGE and immunoblotted with either the anti-myc or anti-V5 antibody.

The immunoprecipitations of endogenous dysbindin-1 and Ku70/80 were performed using the anti-dysbindin-1 antibody linked to gel beads (Seize® Primary Immunoprecipitation Kit, PIERCE). The anti-dysbindin-1 antibody and normal rabbit IgG (control) were coupled to the gel according to the manufacturer's protocol. Lysates from SH-SY5Y cells were mixed with the antibody-coupled gel or with control IgG-coupled gel overnight at 4°C. The gels were washed four times with lysis buffer, and immunoprecipitated proteins were eluted by 100 mM glycine-HCl (pH 2.8). The eluted proteins were analyzed by SDS-PAGE and immunoblotted with anti-Ku70, -Ku80, and -dysbindin-1 antibodies, respectively.

### Immunofluorescence

Cells grown on collagen-coated glass coverslips were washed with PBS and fixed with ice-cold methanol (−20°C) for 20 min. The cells were permeabilized in PBS containing 0.5% (w/v) Triton X-100 for 10 min and blocked in PBS containing 5% normal goat serum (NGS), 0.02% Triton X-100, and 20% glycerol for 1 h at room temperature. Incubations with the primary antibody were performed overnight at 4°C in PBS containing 2% NGS, 0.02% Triton X-100, and 20% glycerol. The cells were washed and then incubated with the appropriate secondary antibodies [Alexa 488 donkey anti-rabbit IgG (1∶1000); Alexa 568 donkey anti-rabbit IgG (1∶1000)] in PBS containing 2% NGS, 0.02% Triton X-100, and 20% glycerol for 1 h. Nuclei were visualized by incubating with TOPRO3 (Invitrogen). After washing with PBS, the cover slips were mounted using Mowiol (Calbiochem, La Jolla, CA). Z-stacks of four to ten images were acquired on a Zeiss LSM510 meta laser scanning confocal microscope (Carl Zeiss, Jena, Germany). Brightest point projections of the Z-stacks were used for image analysis.

### Cell Fractionation

Briefly, cells were supplemented with hypotonic buffer [10 mM Tris-HCl (pH 7.2), 25 mM KCl, 10 mM NaCl, 1 mM MgCl_2_, 0.1 mM EDTA, 1 mM NaF, 1 mM DTT, 0.2 mM PMSF, and 1/1000 volume of protease inhibitor cocktail] and were scraped, passed through a 27-gauge needle ten times, and centrifuged at 100×*g* for 10 min at 4°C. The supernatant was the cytosolic fraction. The resulting crude nuclear pellets were suspended in cell lysis buffer [50 mM HEPES (pH 7.5), 10% glycerol, 0.5% Triton X-100, 150 mM NaCl, 1 mM DTT, 0.2 mM PMSF, and 1/1000 volume of protease inhibitor cocktail] and centrifuged at 16,000×*g* for 60 min at 4°C. The final nuclear pellets were dissolved in RIPA buffer [50 mM HEPES (pH 7.5), 1% Triton X- 100, 0.1% SDS, 150 mM NaCl, 1% deoxycholatic sodium, 1 mM NaF, 1 mM DTT, 0.2 mM PMSF, and 1/1000 volume of protease inhibitor cocktail] and sonicated on ice.

### *In Vitro* Kinase Assay

DNA-PK activity of SH-SY5Y cells transfected with myc-dysbindin-1 was assayed using a synthetic peptide (EPPLSQEAFADLWKK) and [γ-^32^P]-ATP according to the methods of Hosoi [74]. Cell lysate (5 µl) and peptide substrate (5 µg) were mixed in the kinase reaction buffer [20 mM HEPES-NaOH (pH 7.2), 100 mM NaCl, 5 mM MgCl_2_, 50 µM [γ-^32^P]-ATP, 1 mM DTT, and 0.5 mM each of NaF and β-sodium glycerophosphate]. The reaction mixture was incubated at 37°C for 20 min, and the reaction was stopped by addition of 300 mM phosphoric acid. The reaction mixture was spotted onto a P81 paper disk (Whatman), washed in 15% phosphoric acid, and counted in a liquid scintillation counter (Beckman Coulter). Radioactivity was defined as the counts per minute of ^32^P incorporated in the presence of DNA. The counts per minute of ^32^P incorporated in the absence of DNA were used as a control.

DNA-PK activity was assayed using purified GST-dysbindin-1, DNA-PK (Promega), and [γ-^32^P]-ATP. Purified GST-dysbindin-1 (10 µg) and DNA-PK (16 U) were mixed in kinase reaction buffer and incubated at 37°C for 20 min. The reaction was stopped by addition of SDS-sample buffer. To detect phosphorylated proteins, the reaction products were separated on 10% polyacrylamide gels, and the gels were stained with CBB. The gels were then dried, and phosphoproteins were detected by autoradiography using BAS 2500 (Fuji film co.LTD).

### Mn^2+^-Phos-tag SDS-PAGE and Immunoblotting

The samples were prepared from HeLa cells transfected with V5-dysbindin-1 (isoform A, B and C) by immunoprecipitating with the anti-V5 antibody. These samples were divided in half. One half was treated with alkaline phosphatase (AP) (indicated by plus) and the other wasn't (indicated by minus). These samples were separated by Mn^2+^-Phos-tag SDS-PAGE [75] and detected by immunoblotting with the anti-dysbindin-1 antibody according to manufactures' instruction. In the Mn^2+^-Phos-tag SDS-PAGE, Phos-tag acrylamide binds to phosphates in the gel and makes phosphorylated proteins migrate more slowly than unphosphorylated forms of ones.
